# The Amyotrophic Lateral Sclerosis M114T PFN1 Mutation Deregulates Alternative Autophagy Pathways and Mitochondrial Homeostasis

**DOI:** 10.3390/ijms23105694

**Published:** 2022-05-19

**Authors:** Elisa Teyssou, Laura Chartier, Delphine Roussel, Nirma D. Perera, Ivan Nemazanyy, Dominique Langui, Mélanie Albert, Thierry Larmonier, Safaa Saker, François Salachas, Pierre-François Pradat, Vincent Meininger, Philippe Ravassard, Francine Côté, Christian S. Lobsiger, Séverine Boillée, Bradley J. Turner, Danielle Seilhean, Stéphanie Millecamps

**Affiliations:** 1 Institut du Cerveau—Paris Brain Institute—ICM, Inserm, CNRS, APHP, Hôpital de la Pitié Salpêtrière, Sorbonne Université, F-75013 Paris, France; elisa.teyssou@aphp.fr (E.T.); laurachartier@hotmail.fr (L.C.); delphine.roussel@icm-institute.org (D.R.); dominique.langui@icm-institute.org (D.L.); melanie.alb@hotmail.fr (M.A.); francois.salachas@aphp.fr (F.S.); philippe.ravassard@icm-institute.org (P.R.); christian.lobsiger@icm-institute.org (C.S.L.); severine.boillee@sorbonne-universite.fr (S.B.); danielle.seilhean@icm-institute.org (D.S.); 2 The Florey Institute of Neuroscience and Mental Health, University of Melbourne, Parkville, VIC 3052, Australia; pannilage.perera@florey.edu.au (N.D.P.); bradley.turner@florey.edu.au (B.J.T.); 3 Platform for Metabolic Analyses, Structure Fédérative de Recherche Necker, INSERM US24/CNRS UAR 3633, F-75015 Paris, France; ivan.nemazanyy@inserm.fr; 4 Banque d’ADN et de Cellules du Généthon, F-91000 Evry, France; larmonie@genethon.fr (T.L.); saker@genethon.fr (S.S.); 5 Centre de Référence SLA Ile de France, Département de Neurologie, Hôpital de la Pitié-Salpêtrière, APHP, DMU Neuroscience, F-75013 Paris, France; pierre-francois.pradat@aphp.fr; 6 Laboratoire d’Imagerie Biomédicale, Sorbonne Université, INSERM UMRS1146, CNRS UMR7371, F-75013 Paris, France; 7 Northern Ireland Centre for Stratified Medicine, Biomedical Sciences Research Institute Ulster University, C-TRIC, Altnagelvin Hospital, Derry-Londonderry BT47 6SB, UK; 8 Hôpital des Peupliers, Ramsay Générale de Santé, F-75013 Paris, France; vincent.meininger@orange.fr; 9 Institut Cochin, INSERM U1016, CNRS UMR8104, Université de Paris, F-75014 Paris, France; francine.cote@inserm.fr; 10 APHP, Département de Neuropathologie, Hôpital Pitié-Salpêtrière, DMU Neuroscience, F-75013 Paris, France

**Keywords:** ALS, genetics, mutations, alternative autophagy, RAB9, mitochondrial homeostasis, post-mortem spinal cord, lymphoblasts, transgenic mice, NSC-34 cell line

## Abstract

Mutations in profilin 1 (PFN1) have been identified in rare familial cases of Amyotrophic Lateral Sclerosis (ALS). PFN1 is involved in multiple pathways that could intervene in ALS pathology. However, the specific pathogenic role of PFN1 mutations in ALS is still not fully understood. We hypothesized that PFN1 could play a role in regulating autophagy pathways and that PFN1 mutations could disrupt this function. We used patient cells (lymphoblasts) or tissue (post-mortem) carrying PFN1 mutations (M114T and E117G), and designed experimental models expressing wild-type or mutant PFN1 (cell lines and novel PFN1 mice established by lentiviral transgenesis) to study the effects of PFN1 mutations on autophagic pathway markers. We observed no accumulation of PFN1 in the spinal cord of one E117G mutation carrier. Moreover, in patient lymphoblasts and transfected cell lines, the M114T mutant PFN1 protein was unstable and deregulated the RAB9-mediated alternative autophagy pathway involved in the clearance of damaged mitochondria. In vivo, motor neurons expressing M114T mutant PFN1 showed mitochondrial abnormalities. Our results demonstrate that the M114T PFN1 mutation is more deleterious than the E117G variant in patient cells and experimental models and suggest a role for the RAB9-dependent autophagic pathway in ALS.

## 1. Introduction

Amyotrophic lateral sclerosis (ALS), the most frequent adult-onset motor neuron disease, is caused by the progressive degeneration of motor neurons in the spinal cord, brainstem and cortex and is uniformly fatal, usually within five years after disease onset. Most ALS cases are sporadic (SALS), whereas 10% are familial (FALS). A growing number of ALS-linked genes have been identified encoding proteins involved in multiple pathways, including RNA metabolism, DNA repair, cytoskeletal dynamics, vesicular trafficking and protein quality control mechanisms [1].

Mutations in the *PFN1* gene have been identified in some ALS patients. This discovery was based on the whole-exome sequencing (WES) analysis of two distant members from two unrelated large families [2]. After filtering out the most relevant variants, two mutations in the same candidate gene, *PFN1*, were evidenced in these families. The consecutive direct sequencing of *PFN1* in 272 FALS cases confirmed these WES analysis results and identified a total of four potential mutations (C71G, M114T, E117G and G118V) in seven FALS and 2 SALS cases [2]. As the E117G variant was identified in some controls, it was proposed to be less pathogenic [3]. WES analyses were further conducted on large populations of FALS index cases, and controls were supportive of *PFN1* mutation scarcity and modest contribution to ALS heritability [4]. However, several genetic studies reported other PFN1 mutations (A20T, T109M, R136W and Q139L) identified in FALS (0.5–2%) or SALS (0.2–0.8%), confirming that, although rare, PFN1 mutations can cause ALS [5,6,7,8], sometimes presenting as an atypical flail leg syndrome [8]. Moreover, familial segregation could be ascertained for several PFN1 mutations, including C71G, T109M, M114T, E117G and G118V, arguing in favour of their pathogenicity [2,5,7,8,9].

*PFN1* encodes profilin-1, a protein of 140-amino acids with a poly-L-proline binding domain of 6-amino acids and an actin-binding domain of 11 binding sites (all located between amino acids 60–130) [10,11]. Profilin-1 is a nucleotide exchange factor that binds to globular actin (actin monomers) and facilitates its polymerization into actin filaments through a formin-dependent process [12,13].

In addition, functional studies support the pathogenic relevance of PFN1 mutations. Except for the E117G variant, the other PFN1 mutations (C71G, M114T and G118V) impair actin binding, growth cone size and axon outgrowth in neuronal cultures [2]. However, other studies did not confirm a general mechanism for PFN1 mutations that involves actin polymerization defects. Indeed, only the G118V mutation (among the 4 ALS variants initially described) slightly modified the rate of actin polymerization in an in vitro assay measuring actin nucleation and elongation [14]. Moreover, the T109M mutation did not alter actin-binding properties and had no impact on nucleotide exchange activity and actin polymerization [15]. These results suggest that PFN1 mutation pathogenicity could also involve an actin-independent process. A crystallography study showed that PFN1 mutations destabilized and modified the native PFN1 conformation [14]. Thus, it was suggested that mutant PFN1 toxicity could also result from new aberrant interactions preventing its binding to actin or other partners [14]. In line with this hypothesis, PFN1 mutations (A20T, C71G, G118V, M114T, T109M and R136W), and to a lesser extent, the E117G and Q139L variants, led to structural perturbation of the PFN1 protein folded state, increasing its aggregation propensity [16,17]. In addition, in contrast to wild-type (WT) PFN1, ALS mutant (M114T, E117G, G118V) PFN1 failed to bind microtubules and did not stimulate microtubule growth, reinforcing the role of PFN1 in regulating cytoskeleton dynamics that could contribute to motor neuron degeneration [18]. Overexpression of C71G, M114T and G118V mutant forms of PFN1 in primary neurons and neuroblastoma cell lines induced the formation of insoluble ubiquitinated inclusions containing TDP-43, one of the major pathological hallmarks detected in ALS motor neurons [2,19]. This cytoplasmic accumulation of TDP-43 was proposed to result from altered nuclear pore structure and impaired nucleocytoplasmic transport observed for PFN1 mutants (C71G, G118V) [20]. Whether PFN1 mutations contribute to an ALS phenotype through a gain- or a loss-of-function is a source of debate [2,13,21]. However, the evidence supporting a gain-of-function mechanism is far more robust, since the transgenic rodents expressing mutant (C71G, G118V) PFN1 forms elicit motor neuron degeneration and ALS-like phenotypes [22,23,24,25], whereas heterozygous *Pfn1* knockout mice do not show overt motor phenotypes [26].

As profilin-1 interacts with more than 50 ligands, it is involved in multiple cellular pathways that may play a role in ALS pathogenesis [27]. It binds to ATXN2 [28] for which intermediate length polyglutamine expansions have been associated with ALS risk [29] and which is involved in RNA metabolism, regulating P bodies and stress granule formation. Interestingly, recruitment of several PFN1 ALS mutant forms (C71G, M114T and T109M) to arsenite-induced stress granules was impaired compared to WT PFN1 [28]. Another PFN1 binding partner is the valosin-containing protein (VCP, also known as transitional endoplasmic reticulum ATPase) [30], which is involved in multiple cellular pathways, including the maturation of autophagosomes formed to clear ubiquitinated proteins [31]. Mutations in the *VCP* gene have been identified in FALS [32] and some of these mutations impaired autophagosome formation [33]. Another potential PFN1 partner is PTEN [34], Phosphatidylinositol 3,4,5-trisphosphate 3-phosphatase and dual-specificity protein phosphatase, which is involved in the regulation of macroautophagy by dephosphorylating PIP3 (to PIP2), thus limiting the activation of the AKT and mTOR pathway [35].

In the present study, we hypothesized that PFN1 could play a role in autophagy pathways, and that PFN1 ALS mutations could disrupt this function. To explore this hypothesis, we analysed a large population of ALS patients to identify PFN1 mutations and characterise their effects on autophagic marker expression in patient lymphoblasts, as well as in PFN1 expressing models.

## 2. Results

### 2.1. PFN1 Mutations Are Rare in French ALS Patients

We first aimed to confirm the contribution of the *PFN1* gene (NM_005022.3) to ALS by performing genetic analyses of large cohorts, including a total of 750 French ALS patients (with 150 FALS and 600 SALS) devoid of mutations in *C9orf72*, *SOD1*, *TARDBP*, *FUS* or *UBQLN2*. We identified 2 *PFN1* missense substitutions located in exon 3: the c.341T>C, p.Met144Thr (M114T) mutation that segregated with the disease in two affected FALS siblings (frequency of 0.6%) and the double nucleotide substitution: c.350_351AA>TG, pGlu117Gly (E117G) variant in two SALS cases, including one autopsied case (frequency of 0.3%) and 1 control (out of 500) of 65 years at blood sampling (frequency of 0.2%). Both mutations were already identified in the original discovery of PFN1 mutations in ALS [2]. The M114T mutation was not listed in the Exome variant server NHLBI GO Exome Sequencing Project (ESP), the ExAC (Exome Aggregation Consortium) and gnomAD (Genome Aggregation Database) browsers, whereas the E117G variant had a frequency of 0.07% and 0.05% in European Non-Finnish populations from the ExAC and gnomAD browsers, respectively. These mutations affected amino acids that were conserved in mammals and were located close to a possible actin-binding site (concerning amino acids 119–123). Patient clinical data are presented in Table S1. Interestingly the autopsied patient carrying the E117G variant also had a variant in the *ANG* gene (K17I) that was previously reported [36,37]. Neuropathological analyses performed on this patient showed the presence of intranuclear neuronal inclusions, immunopositive for smooth muscle alpha-actin (smA), in areas apparently spared by the neurodegenerative process [36]. Further investigations performed in this patient for PFN1 immunostaining did not reveal any PFN1-positive deposits, either colocalized with smA inclusions, or at some distance, throughout the brain and spinal cord, including neurons with TDP-43 inclusions (data not shown).

### 2.2. PFN1 Protein Levels Are Decreased in M114T Patient Lymphoblasts

**Figure 1:**
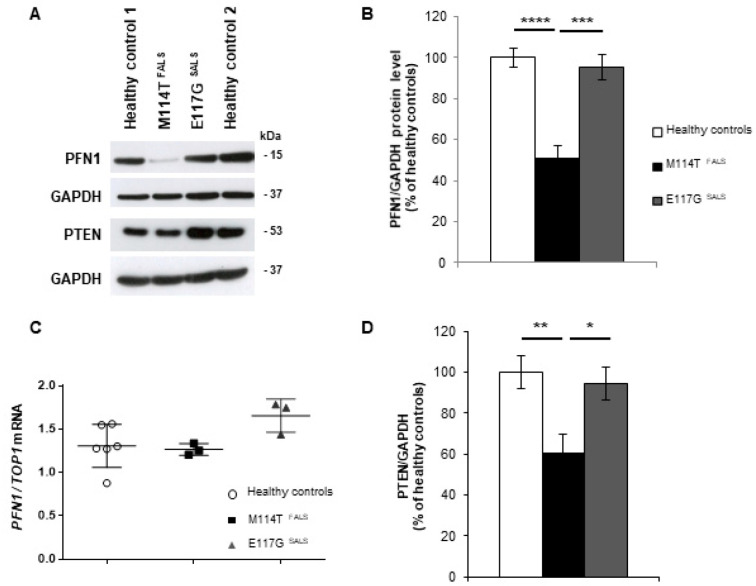
PFN1 and PTEN protein levels are decreased in M114T lymphoblasts. (**A**) Immunoblot analysis of protein extracts from lymphoblasts of healthy controls and ALS patients carrying the M114T mutation or E117G variant using anti-PFN1, PTEN and GAPDH antibodies. (**B**) Densitometry analyses of PFN1 and (**D**) PTEN protein levels for healthy controls (white), M114T patient (black) and E117G patient (grey) standardized to GAPDH levels and represented as a percentage of the healthy controls. Results are means ± standard errors of the mean (SEM) for at least 7 independent experiments. (**C**) Semiquantitative Reverse Transcription PCR (RT-PCR) for *PFN1* mRNA extracted from healthy controls (white circles), M114T (black squares) and E117G (grey triangles) patient lymphoblasts. The levels of *PFN1* mRNA were related to that of DNA topoisomerase 1 (*TOP1*). Results are means ± standard errors of the means (SEM) of 3 independent experiments. * *p* < 0.05; ** *p* < 0.01; *** *p* < 0.005; **** *p* < 0.001.

To study the functional impact of the mutations we identified, lymphoblastoid cell lines were established from lymphocytes of these ALS patients carrying the M114T mutation or E117G variant and compared to lymphoblastoid cells established from two healthy controls. We first studied PFN1 protein levels in these lymphoblasts. Western blot analyses showed a 50% loss of PFN1 expression in the M114T patient lymphoblasts, compared to healthy controls (Figure 1A,B). In contrast, the level of expression of PFN1 was not modified in lymphoblasts with the E117G variant. To determine whether PFN1 protein reduction was due to a decrease in PFN1 gene expression, we extracted mRNA from these lymphoblasts and performed semi-quantitative RT-PCR for PFN1 and DNA topoisomerase 1 (TOP1) as a standard gene. Results showed that this loss was not due to a decrease in PFN1 mRNA expression in M114T lymphoblasts, as no difference in the quantity of PFN1 cDNA was observed between mutant and control lymphoblasts (Figure 1C).

Low M114T protein levels could result from protein instability, as previously observed for this mutant form in transfected neuronal cells [14]. We performed time-course experiments to measure the rate of protein degradation in patient lymphoblasts after cycloheximide treatment to inhibit translation: M114T PFN1 protein had a significantly faster turnover rate than the wild-type (WT) and E117G forms (Figure S1). In contrast, there was no significant difference between the turnover rates of WT and E117G forms of PFN1. These findings support that the loss of M114T mutant PFN1 protein in patient lymphoblasts may result in part from protein instability.

### 2.3. Autophagy-Linked PTEN Shows Decreased Levels in M114T Patient Lymphoblasts

As modifications of PFN1 levels were previously shown to impact PTEN levels in breast tumour cells [34,38], we evaluated the protein levels of PTEN in PFN1 patient lymphoblasts by Western blot analysis. PTEN levels were reduced by 40% in patient cells carrying the M114T mutation compared to the healthy control (Figure 1D). Since PTEN is involved in the regulation of the mTOR-mediated autophagy pathway [35], we explored the autophagy pathway in mutant PFN1 lymphoblasts.

### 2.4. The Alternative Autophagy Pathway Is Deregulated in M114T Patient Lymphoblasts

Autophagosomes assembled during macroautophagy are of two types [39,40]. In the Atg5/Atg7-dependent conventional autophagy pathway, they are composed of the LC3II protein formed by the conjugation of cytosolic LC3I with phosphatidylethanolamine and are thought to derive from endoplasmic reticulum membranes [41]. In the proposed Atg5/Atg7-independent autophagy process, they are composed of the small GTPase Ras-related protein Rab-9A (RAB9) and could derive from trans-Golgi and late endosome membranes [39,40,42]. Both macroautophagy types coexist in the same cells [42]. During conventional macroautophagy, levels of LC3II are modulated, firstly increased at the induction step and then decreased by lysosomal degradation [43,44,45]. In the alternative pathway, as RAB9 is thought to mediate a similar role to LC3II in conventional autophagy [39], we assumed that the levels of RAB9 may be changed during the occurrence and/or deregulation of the alternative pathway.

**Figure 2:**
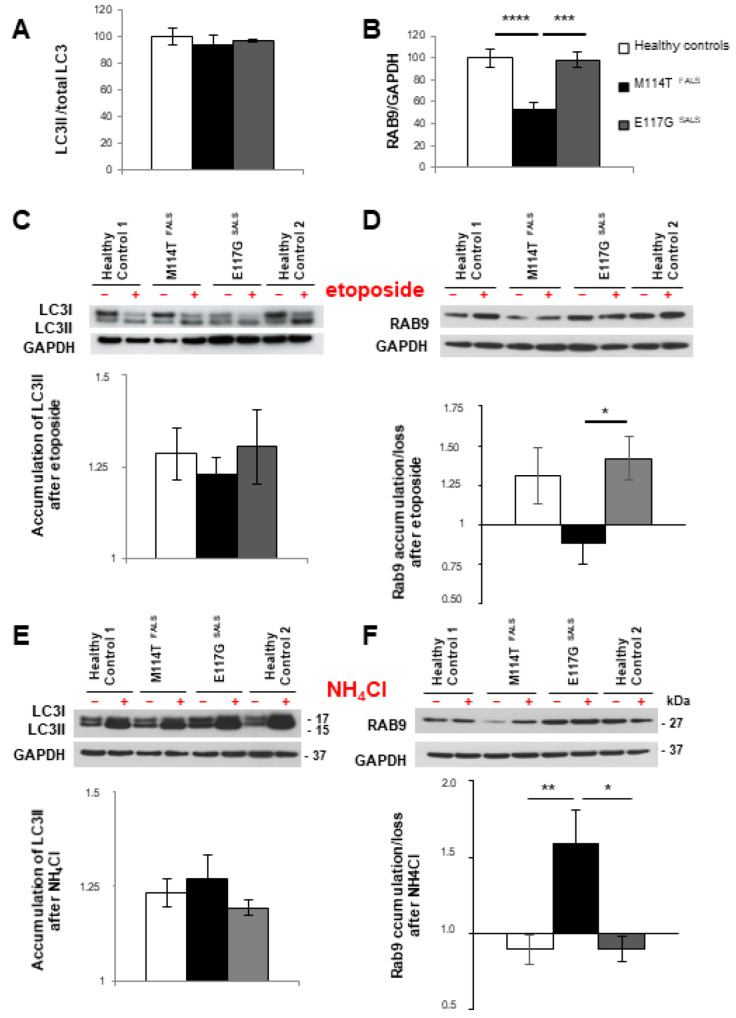
Deregulation of macroautophagy in M114T lymphoblasts. (**A**–**F**) Immunoblot analysis of protein extracts from lymphoblasts of healthy controls (white) and ALS patients carrying the M114T (black) and E117G (grey) PFN1 mutations. (**A**) Densitometry analyses show the amount of LC3II to total LC3 or (B) RAB9 levels standardized to GAPDH expression. Protein levels are represented as a percentage of the healthy controls. (**C**,**D**) Patient lymphoblasts were treated (+) or not (−) with etoposide autophagy inducer or (**E**,**F**) NH_4_Cl lysosomal inhibitor. (C, E, lower panel) Accumulation of LC3II and (D, F, lower panels) RAB9 after these treatments were evaluated by densitometry and presented as fold of induction. Results are means ± SEM for 3 to 8 independent experiments. * *p* < 0.05; ** *p* < 0.01; *** *p* < 0.005; **** *p* < 0.001.

To determine whether macroautophagy processes were modified in lymphoblasts with PFN1 mutations, we compared the levels of LC3II and RAB9 before and after treatments modulating autophagic flux. Before treatment, the amount of LC3II was not different between the patient and control lymphoblasts (Figure 2A). In contrast, RAB9 protein levels were decreased by 50% in M114T patient lymphoblasts compared to control and E117G lymphoblasts (Figure 2B). This loss was specific for RAB9 protein levels as *RAB9A* mRNA levels remained unchanged (Figure S2).

Loss of RAB9 was shown to prevent etoposide-induced alternative autophagy in embryonic fibroblasts [42]. Etoposide can also activate canonical autophagy by increasing ATG5 activity [46]. Thus, we used etoposide treatment to compare, between patient and control lymphoblasts, the capacity of both macroautophagy processes to be stimulated. After etoposide treatment, LC3II levels were similarly increased in control and patient lymphoblasts with M114T or E117G PFN1 mutations (Figure 2C). However, etoposide treatment resulted in increased RAB9 levels in control and E117G SALS lymphoblasts but did not modify RAB9 levels in M114T PFN1 lymphoblasts (Figure 2D). This result suggests disturbances of alternative autophagy in M114T lymphoblasts.

Then, we used ammonium chloride (NH_4_Cl), a lysosomal inhibitor that prevents lysosome acidification required for autophagosome-lysosome fusion [47], and compared the impact of this treatment on autophagic marker levels in patient and control lymphoblasts. LC3II levels similarly accumulated after the NH_4_Cl treatment in patient and control cells (Figure 2E). In contrast, an increase of RAB9 protein levels was observed only for the patient carrying the M114T mutation compared to other groups (Figure 2F), suggesting that RAB9 protein loss in M114T lymphoblasts could result, at least in part, from its increased lysosomal degradation.

### 2.5. Mitochondrial Homeostasis Is Misregulated in M114T Lymphoblasts

**Figure 3:**
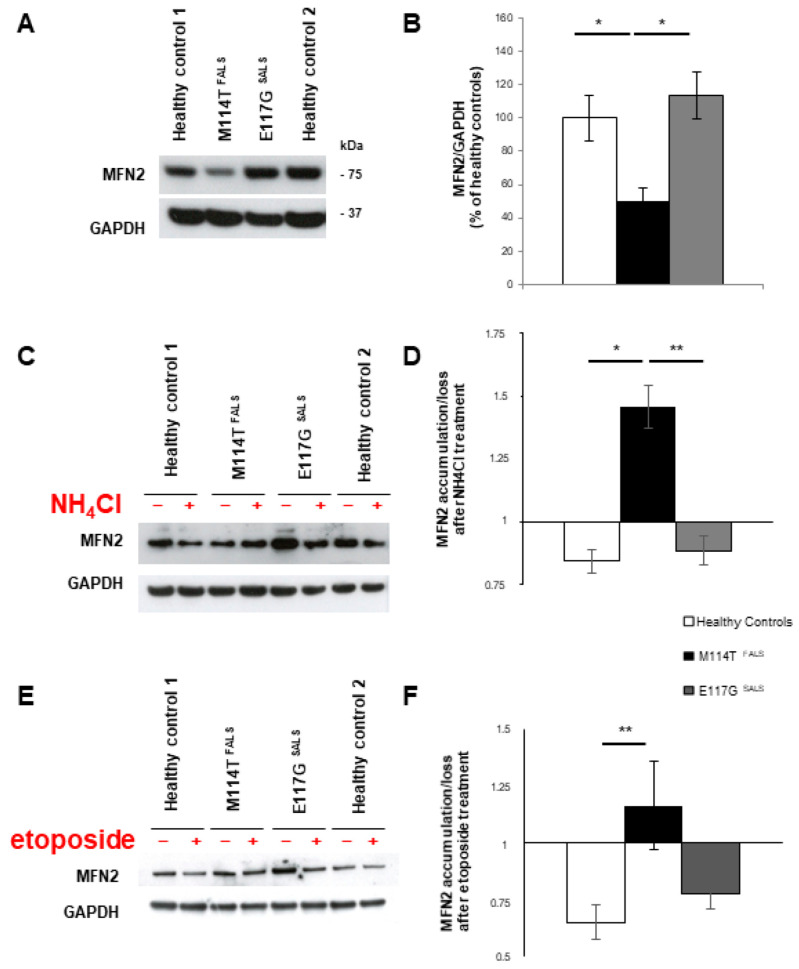
Deregulation of MFN2 mitochondrial marker in M114T patient lymphoblasts. (**A**) Anti-MFN2 was used to quantify mitochondrial levels in lymphoblast protein extracts. (**B**) Densitometry analyses of MFN2 protein levels for healthy controls (white), M114T patient (black) and E117G patient (grey), normalized to GAPDH levels and presented as a percentage of healthy controls. (**C**,**D**) NH_4_Cl or (**E**,**F**) Etoposide treatments were used to modulate the RAB9-mediated alternative mitophagy. (**D**) Accumulation/loss of MFN2 after NH_4_Cl treatment and (**F**) after etoposide treatment were represented for healthy controls (white), M114T patient (black) and E117G patient (grey). Results are means ± SEM of 4 to 8 independent experiments. * *p* < 0.05; ** *p* < 0.01.

Experiments previously performed in reticulocytes or in HeLa cells supported the role of RAB9-linked autophagosomes in mitophagy, a quality control mechanism that removes damaged mitochondria [48,49]. We hypothesized that modification of alternative autophagy observed in M114T patient lymphoblasts could have an impact on overall mitochondrial levels. First, we compared protein levels of the mitofusin 2 (MFN2) mitochondrial marker in patient and control lymphoblasts, which were decreased in the M114T PFN1 patient (Figure 3A,B). As observed for RAB9 (Figure 2F), MFN2 protein loss was also restored in M114T lymphoblasts upon lysosomal degradation blockade with NH_4_Cl (Figure 3C,D). Moreover, activation of autophagy pathways using etoposide failed to reduce the content of mitochondria in these cells, although this was observed in controls and SALS with E117G variant (Figure 3E,F). These results were confirmed after measuring cytochrome C (CYCS) mitochondrial protein levels and quantification of mitochondrial ATP production: both were decreased in M114T patient lymphoblasts (Figure S3A–C). Taken together, these results suggest that mitochondrial clearance was promoted in these M114T patient lymphoblasts.

### 2.6. RAB9 and Mitochondria Levels Are Reduced in Cells Expressing a PFN1 M114T Transgene

**Figure 4:**
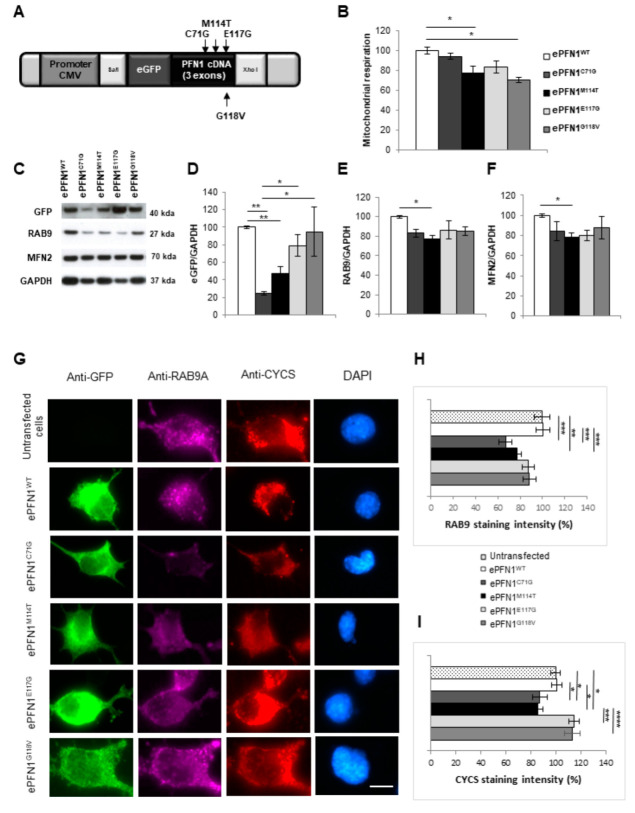
Deregulation of autophagic and mitochondrial markers in M114T transfected cells. (**A**) Schematic representation of plasmid constructs to overexpress PFN1 fused to eGFP gene. (**B**) Mitochondrial respiration was measured in HEK293T expressing ePFN1^WT^ (white), ePFN1^C71G^ (dark grey), ePFN1^M114T^ (black), ePFN1^E117G^ (light grey) and ePFN1^G118V^ (grey) plasmid constructs using the MTT assay. To facilitate data interpretation, the mitochondrial respiration of PFN1^WT^ was adjusted to 100%. (**C**) Immunoblots were performed using anti-GFP, RAB9, MFN2 and GAPDH antibodies on HEK293T protein extracts. (**D**) Densitometry analyses of eGFP, (**E**) RAB9 and (**F**) MFN2 protein levels in HEK293T cells transfected with the 5 plasmid constructs were normalized to those of GAPDH and are presented relative to those of ePFN1^WT^. Results are means ± SEM for at least 9 independent experiments. (**G**) NSC-34 were transfected with eGFP-PFN1 constructs expressing WT or various mutant forms of PFN1 (C71G, M114T, E117G or G118V) and triple immunofluorescence was performed to detect mitochondria (using CYCS in red) and RAB9A (in far-red) in cells with and without expression of eGFP tagged PFN1 constructs (in green). Nuclei are stained with DAPI (blue). Bar is 10 µm. (**H**) Fluorescent signal intensities of RAB9A and (**I**) CYCS were measured in cells expressing the eGFP-PFN1 constructs and are presented relative to those recorded in untransfected cells. Results are means ± SEM for 3 independent experiments with 20 to 70 transfected cells recorded for each plasmid condition. * *p* < 0.05; ** *p* < 0.01; *** *p* < 0.005; **** *p* < 0.001.

To confirm the observations made in lymphoblasts, we constructed five plasmids expressing WT, M114T or E117G mutants or other known mutants (C71G and G118V). PFN1 cDNAs were linked to an enhanced green fluorescent protein (eGFP) tag under the control of the strong cytomegalovirus (CMV) promoter (Figure 4A). These five plasmid constructs were first tested after transfection of HEK293T cells to measure the levels of eGFP-tagged PFN1. As observed in patient lymphoblasts, the PFN1 protein levels (human transgene measured through eGFP expression) were lower for M114T than for the WT, E117G and G118V constructs (Figure 4C,D). A similar lower PFN1 expression was detected for the C71G mutant (Figure 4C,D).

To analyse the pathogenic role of the M114T mutation on autophagy processes and mitochondrial homeostasis, RAB9 and MFN2 protein levels were also quantified. A slight decrease of both RAB9 (20%) and MFN2 (26%) was determined in the ePFN1^M114T^ transfected cells compared to cells transfected with the PFN1^WT^ transgene (Figure 4E,F). The impact of PFN1 mutations was also analysed on the mitochondrial respiration of HEK293T cells using the modified colorimetric MTT assay [50]. There was a slight decrease in the mitochondrial respiration measured by this test that reached statistical significance for cells expressing ePFN1^M114T^ and ePFN1^G118V^, compared to cells expressing PFN1^WT^ (Figure 4B). Similar results were obtained after the transfection of three other plasmids expressing WT or mutant (M114T or E117G) PFN1 cDNA linked to a hemagglutinin (HA) tag under the control of the ubiquitously expressed human Ubiquitin c (UBC) promoter that were designed for lentiviral transgenesis (Figure S4).

These results were confirmed in the NSC-34 motor neuron cell line. After transfection with eGFP-PFN1 transgenes, RAB9 expression and mitochondrial content and distribution were compared by immunofluorescence using anti-RAB9A and anti-CYCS antibodies, respectively (Figure 4G). We observed a reduction in RAB9 immunostaining and mitochondrial labelling in NSC-34 expressing ePFN1^M114T^, compared to ePFN1^WT^ and untransfected cells (Figure 4G–I). A similar downregulation of RAB9 and mitochondrial staining was recorded for the ePFN1^C71G^ construct. Altogether, these data obtained in cellular models confirmed the results obtained in patient lymphoblasts.

### 2.7. Expression of PFN1^M114T^ Mutation in Motor Neurons In Vivo Leads to Abnormal Mitochondrial Distribution

**Figure 5:**
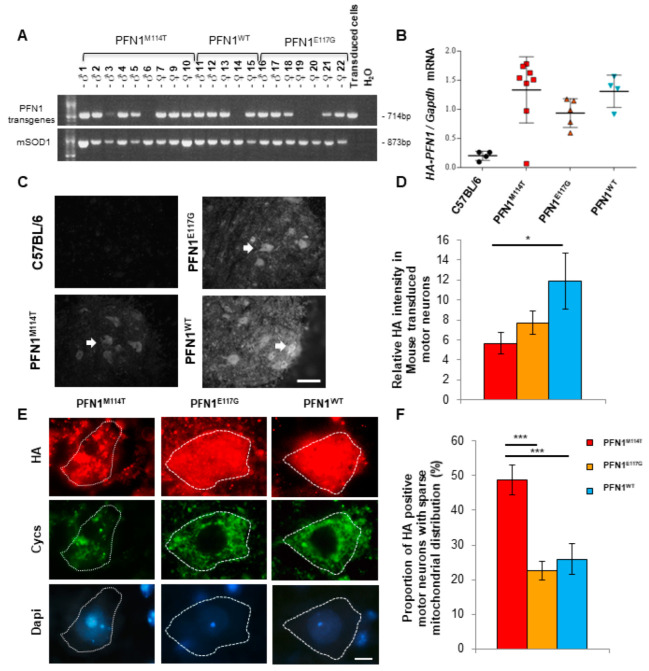
Instability of M114T PFN1 protein and histological analyses in PFN1 mouse spinal cord. (**A**) PCR were performed on DNA extracted from tail fragments with primers specific for PFN1 transgene and Sod1 fragment as a marker of DNA quality. (**B**) Semi-quantitative mRNA determination for HA-tagged PFN1 transgenes related to that of Gapdh for C57Bl/6 (black circles), PFN1^M114T^ (red squares), PFN1^E117G^ (orange triangles) and PFN1^WT^ (blue inverted triangles) mice. Note that mice with no transgene detected in A (mice 6, 14, 19, 20) were not included in B. (**C**) Immunofluorescence analysis using anti-HA antibody was performed on lumbar spinal cord sections to detect the transgene in the C57BL/6, PFN1^M114T^, PFN1^E117G^ and PFN1^WT^ mice. White arrows point to HA positive motor neurons. Bar: 40 µm. (**D**) The relative intensity of HA fluorescence measured in transduced motor neurons is lower in PFN1^M114T^ than in PFN1^E117G^ and PFN1^WT^. (**E**) Double immunofluorescence staining was performed on lumbar spinal cord sections using anti-HA antibody to detect PFN1 transgene (in red) and Cycs (in green). Dapi stained large nuclei with intense nucleoli in motor neurons. Mitochondria were sparse in motor neurons overexpressing the PFN1^M114T^ transgene. Bar: 10 µm. (**F**) Proportion of HA positive motor neurons with sparse mitochondrial distribution in lumbar spinal cord for PFN1^M114T^ (red), PFN1^E117G^ (orange) and PFN1^WT^ (blue) groups of mice. * *p* < 0.05, *** *p* < 0.005.

To further compare the pathogenicity of M114T and E117G mutations on motor neurons in vivo, we used lentiviral constructs to generate three novel mouse models expressing wild-type (WT) or mutant (M114T or E117G) PFN1 by lentiviral transgenesis [51]. This approach was similarly efficient for the three PFN1 transgenes: 80% (WT), 70% (E117G) and 89% (M114T) of the progeny carried the transgenes as assessed by PCR (Figure 5A). The presence of the mutations was verified by Sanger sequencing in all positive mice. Using lentiviral transgenesis, every mouse is unique. We did not breed the F0 mice to establish a line and chose to directly analyse them as previously recommended [51], which considerably reduced the number of mice used in this study. Several behavioural tests were performed weekly from the age of 3 months to evaluate the motor performance of these PFN1^WT^, PFN1^M114T^ and PFN1^E117G^ transgenic mice compared to C57BL/6 control mice. These analyses did not show motor phenotypes in these mice (Figure S5).

To determine whether PFN1 mice presented abnormalities on electromyography (EMG) recordings resembling those observed for ALS patients and mutant SOD1 transgenic mice [52,53], EMG recordings were performed in the gastrocnemius muscle at 11 and 17 months for the four groups of mice. Spontaneous activities (fasciculations and/or fibrillations, Figure S6A,B) were detected in the gastrocnemius muscles of all PFN1^M114T^ mice (100%) at 17 months of age, whereas they were not detected at 11 months. The severity of these spontaneous activities was higher for the PFN1^M114T^ mice than for the other groups (Figure S6C). No difference between the four groups was observed for the evoked potentials (determined using Compound Muscle Action Potential and distal motor latency) at 11 and 17 months (Figure S6D,E).

All the mice were euthanized at 17 months and tissues were collected. Mouse spinal cord sections were subjected to immunofluorescence analysis using an anti-HA antibody to detect transgenic PFN1. HA-tagged transgenic PFN1 was detected in motor neurons and their surrounding cells in PFN1^WT^, PFN1^M114T^ and PFN1^E117G^ mice but not in C57BL/6 control mice (Figure 5C). However, in line with data in cell cultures showing instability of the PFN1^M114T^ protein, a difference in transgene expression intensity was observed between the groups of mice. It was lower for PFN1^M114T^ mice than the PFN1^WT^ group, with medium intensity for PFN1^E117G^ (Figure 5D). In contrast, no decrease in HA-tagged PFN1 mRNA levels was observed in the spinal cord of the M114T mice compared to WT or E117G groups using HA-tagged PFN1 specific primers (Figure 5B).

To determine whether PFN1 mutant expression impaired mitochondrial homeostasis in vivo, the mitochondrial distribution was compared in motor neurons expressing PFN1 transgenes using double immunofluorescence with anti-HA and anti-cytochrome C antibodies. Mitochondria were distributed homogeneously in PFN1^WT^ and PFN1^E117G^ expressing motor neurons (Figure 5E). In contrast, in motor neurons expressing PFN1^M114T^, the mitochondria were sparse, swollen and enlarged compared to those observed in motor neurons expressing other PFN1 transgenes (Figure 5E). Quantitative analyses confirmed that mitochondrial sparse distribution was more frequently observed in motor neurons expressing PFN1^M114T^ (Figure 5F).

Mitochondrial abnormalities were confirmed with electron microscopy observations of motor neuron axons in lumbar ventral roots. Elongated mitochondria with dark matrix and well-defined cristae structures were mainly observed in non-transgenic, PFN1^WT^ and PFN1^E117G^ mice (Figure S7A,B). Several mitochondrial abnormalities were observed in all transgenic mice, including the presence of membrane vesicles in close apposition to the outer mitochondrial membrane, as well as mitochondria engulfing cytosolic components, as illustrated for PFN1^WT^ (Figure S7C) or PFN1^E117G^ (Figure S7D). In PFN1^M114T^ mice, additional mitochondrial degenerative changes included clear matrix with remnants of cristae membranes (Figure S7E–G), various engulfed materials (Figure S7E,F) or aberrant shapes (Figure S7G). After quantification, mitochondrial abnormalities were more frequently observed in PFN1^M114T^ mice (Figure S7H).

## 3. Discussion

We identified two PFN1 variants in our cohort of 150 FALS and 600 SALS patients: the M114T mutation has a frequency of 0.6% in FALS and the E117G variant was present in 0.3% of SALS and 0.2% of controls. These frequencies were similar in other populations: they were reported to be 0.7% of FALS for M114T and 0.3% of SALS and 0.04–0.3% of controls for the E117G variant, respectively [2,3,54]. The latter was suggested to be a less pathogenic variant according to both frequency data reported in control subjects and ALS cases, and functional studies [2,3,5,7,54,55,56] and could rather be a susceptibility variant for ALS. In accordance with this hypothesis, one of the SALS patients with the E117G variant we identified also carried another variant (K17I) in ANG (encoding angiogenin), another ALS related gene [57]. We cannot exclude that the co-occurrence of both variants led to an ALS phenotype in this patient. Indeed a growing number of studies have reported the presence of several mutations in different ALS related genes, arguing in favour of the oligogenic inheritance of ALS in some cases [58,59,60,61].

The quantitative analysis we performed on patient lymphoblasts further showed that the M114T mutant protein level was reduced, despite maintaining PFN1 mRNA gene expression and that the turnover of this mutant form of protein was accelerated. These results were confirmed after overexpression of various PFN1 constructs in cell lines: the protein levels of the tagged PFN1^M114T^ were lower than the levels of PFN1^WT^ and PFN1^E117G^, confirming that the PFN1^M114T^ form was less stable. In mouse spinal cord tissue, expression of PFN1^M114T^ was also lower than for the other transgenes at the protein level, although it remained unchanged at the mRNA level. These results suggest that the M114T mutant form is more unstable than the native or E117G mutant proteins. This is in accordance with the faster turnover rate observed for this PFN1 mutant form in neuronal cells and the crystallography experiments showing that M114T protein instability may be linked to the large cavity induced by this mutation close to the core of the protein [14]. A similar protein instability has already been reported for the C71G mutant form of PFN1 in patient lymphoblasts and cell lines [13]. Misfolded and aggregated forms of PFN1 observed with C71G, M114T and G118V mutants suggest that modification of stability of these PFN1 mutants increased their aggregation propensity [2,14].

A previous study detailed the neuropathological analysis of three autopsied ALS patients (two carrying the E117G and one with the Q139L PFN1 variant). No PFN1 immunopositivity was seen in any of the CNS regions examined [7]. The neuropathological examination we made for the SALS case carrying both the E117G PFN1 and K17I ANG variants confirmed the absence of PFN1 deposits. Moreover, nuclear smA positive inclusions we previously described for this patient [36] were all negative for PFN1. Altogether, these results revealed no evidence of PFN1 accumulation in PFN1 patient lymphoblasts and post-mortem tissues. No positive PFN1 inclusions were either previously detected in any other patients with ALS, including those with a mutation in *C9orf72*, *SOD1*, *FUS* or *UBQLN2* [55].

In cancer cells, overexpression of PFN1 has been shown to impact PTEN levels, preventing its degradation by the proteasome [34,38]. Our experiments performed in ALS patient lymphoblasts showed that, similarly to PFN1 levels, PTEN was decreased in lymphoblasts with the M114T mutation, suggesting that PTEN loss could result from PFN1 protein destabilization. PTEN is a key regulator of autophagy. Indeed, it prevents AKT phosphorylation and activation of mTOR, an inhibitor of autophagy, thereby activating the autophagy pathway [35]. Therefore, a decrease in PTEN levels is expected to impact autophagy.

Analyses of autophagic markers we performed in patient lymphoblasts, revealed various modifications in M114T cells, all affecting the RAB9-dependent alternative autophagic process: M114T lymphoblasts showed decreased RAB9 levels at basal state, no RAB9 increase after etoposide treatment and increased RAB9 accumulation after lysosomal blockade, suggesting that this RAB9 loss was due, at least in part, to its lysosomal degradation. Altogether, these observations converged to the hypothesis that the alternative autophagy pathway could be already activated in M114T lymphoblasts. However, we cannot exclude that this RAB9 loss could also be consecutive to other cellular defects and could have by itself, directly or indirectly, activated the alternative autophagy process. Whether these alterations are due to a direct effect of mutant PFN1 or are a consequence of undetermined cellular disturbances, remains to be explored.

The precise role of alternative autophagy is unclear. RAB9-linked autophagosomes have been proposed to contribute to mitophagy, a quality control mechanism that removes damaged mitochondria to prevent their accumulation, notably within neurons [48,49]. Indeed, depolarized or damaged mitochondria after fission events are eliminated through a process that involves PINK1 (PTEN-induced putative kinase 1), which accumulates on the mitochondrial outer membrane and recruits PARK2 E3 ubiquitin ligase. During this process, ubiquitination of many mitochondrial outer membrane proteins marks mitochondria for degradation [62]. Mitophagy can also be induced by cellular stress and, in this case, involves conventional and alternative macroautophagy pathways [49]. Since mitophagy was shown as severely compromised in RAB9 knockdown cells, mitophagy was suggested as being primarily due to the alternative autophagy pathway [48,49]. Experiments we performed in M114T PFN1 lymphoblasts showed a decrease in basal levels of MFN2, a GTPase localized in the outer membrane of mitochondria and CytC, an electron-transporting protein between complexes III (CytC reductase) and IV (CytC oxidase) of the mitochondrial respiratory chain. In addition, MFN2 levels paralleled those of RAB9 in M114T lymphoblasts since they were not increased after etoposide treatment and were restored after NH_4_Cl lysosomal inhibition.

We used overexpression models to compare the direct impact of PFN1 mutations on RAB9 and mitochondrial protein levels in motor neuron-like cells without any contribution of the patient’s genetic background. Our immunofluorescence and Western blot experiments showed evidence for reduced mitochondrial content in NSC-34 and HEK293T cells transfected with PFN1^M114T^: they presented decreased levels of MFN2 and CYCS, associated with reduced mitochondrial respiration. These results confirmed that M114T mutation caused deregulation of mitochondrial homeostasis.

Altogether, these results in M114T patient lymphoblasts and cells overexpressing M114T PFN1 are in favour of a mechanism involving overstimulation of the alternative autophagy pathway mediating mitochondrial clearance in these cells, resulting in decreased mitochondrial abundance. This is also consistent with the decrease in ATP production observed in M114T lymphoblasts. Another hypothesis would be that the mitochondrial defects could be the primary event occurring in M114T lymphoblasts, driving an energetic breakdown and cellular stress that would consequently trigger alternative autophagy stimulation.

This hypothesis is supported by our results in transgenic mice. Indeed, we observed in vivo, that the expression of PFN1^M114T^ in mouse motor neurons triggered abnormal mitochondrial morphology and distribution. Mitochondrial homeostasis was shown to be defective in various mouse models of ALS. Swollen circular mitochondria have been observed in both motor neuron axons and cell bodies in mutant SOD1 transgenic mice at pre-symptomatic disease stages, with increased severity with disease progression [63]. In this model, increased mitochondrial volume was suggested to arise from either mitochondrial swelling due to defects in ion homeostasis, altered mitochondrial fission/fusion dynamics, or defective autophagy resulting in the inability to clear large or swollen mitochondria [64]. These results support the view that mitochondrial defects are deleterious for motor neuron survival during the ALS neurodegenerative process.

Ultrastructural mitochondrial alterations have already been reported in motor neuron axons of transgenic mice expressing the G118V mutant form of PFN1 [22]. In these mice, mitochondria were fragmented with disorganized cristae at end-stage of clinical disease. The authors suggested that the actin cytoskeleton abnormalities caused by this PFN1 mutant possibly impaired mitochondrial dynamics and induced a Wallerian-like degeneration [22]. Indeed, actin polymerization/depolymerization was proposed to modulate fission/fusion mitochondrial balance [65]. In another study, aberrant ultrastructural defects in mitochondria were obvious very early on in PFN1^G118V^ mice, prior to any neuronal defects, specifically in upper motor neuron axons [66]. These authors proposed that mitoautophagy (a self-clearance mechanism of mitochondria that did not require autophagosomes) was implicated in these mutant PFN1^G118V^ mice.

Our results in cell lines showed that the M114T defects (impaired mitochondrial respiration, decreased RAB9, MFN2 levels or CYCS staining intensity) are shared by other PFN1 mutants (including the C71G and G118V). In PFN1^M114T^ mice, we observed mitochondrial structural defects without any motor phenotype, suggesting mitochondrial defects occurred early in the motor neuron degenerating process.

Other transgenic mouse models expressing strong levels of C71G or G118V mutant PFN1, under the control of neuronal Thy1.2 or PrP promoters, showed strong motor phenotypes and decreased survival, and loss of motor neurons in the spinal cord, resembling ALS [22,23,24,25]. We did not observe a strong disease phenotype using our lentiviral transgenesis approach based on the expression of PFN1 under the control of the human ubiquitin C promoter. This promoter was shown to drive transgene expression mainly in neurons, although some neurons were not targeted [67]. Our immunofluorescence results showed that the promoter we used drove expression in motor neurons, but the level could be too low to induce the rapid phenotype previously observed for the other mouse lines expressing 4 to 5 times more PFN1 transgene levels than endogenous *Pfn1* [22,23]. Nevertheless, we observed an increased number of spontaneous activities (presence of fasciculations and/or fibrillations) after EMG recordings in our PFN1^M114T^ mice. Such abnormalities were observed at disease onset in patients, at early disease stages in mutant SOD1 ALS mouse models [52,53,68] and at the disease end-stage in transgenic G118V mutant PFN1 mice [22].

Overall, our results in patient lymphoblasts and overexpression systems in vitro highlight a new role of PFN1 in autophagy regulation and suggest that the M114T ALS mutant acts by disrupting this pathway and leads to abnormal mitochondrial clearance. Our findings in vivo suggest that PFN1^M114T^ motor neurons were vulnerable with early mitochondrial defects that could trigger clinical disease at later stages. Our results propose a link between RAB9-mediated alternative autophagy and mitochondrial clearance in cells expressing the PFN1^M114T^ mutant. In all our experiments in vitro and in vivo, the M114T mutation was more deleterious than the E117G variant. As the actin network is involved in the formation of LC3II-derived autophagosomes and their fusion with lysosomes, and as the deregulation of actin polymerization is detrimental for autophagosome formation/degradation [69,70], the effect we observed on autophagy pathways in M114T lymphoblasts could be a consequence, at least in part, to a defect in actin polymerization regulation. Whether our observations are linked to actin cytoskeletal alterations, need further investigations.

Several ALS related-genes, including the oldest (*SOD1*) to the most recently identified (*SQSTM1, OPTN* and *TBK1*) ones, have been linked to mitochondrial homeostasis [62]. For example mutant SOD1 accumulates on the mitochondrial outer membrane [71] and provokes defects in the mitochondrial respiratory chain activities [72]. After being phosphorylated by TBK1, p62 (encoded by *SQSTM1*) and OPTN are both recruited to damaged mitochondria to target them to autophagosomes [73]. Thus, mitochondrial regulation seems to be shared by several ALS-related genes and could play a crucial role during the disease process (by causing, for example, bioenergetic failure, decreased ATP availability for axonal transport, and immobilization of mitochondria facilitating their removal by autophagy) [74]. Besides accumulating evidence emphasizing the importance of defects in ribostasis and proteostasis in ALS, our study points to defects in “mitochondriostasis” as a possible converging pathway that would, therefore, deserve further attention.

## 4. Materials and Methods

### 4.1. Genotyping

Patient DNA cohorts from 150 FALS and 600 SALS, including 79 autopsied patients, were analysed in this study. ALS was diagnosed according to the El Escorial criteria [75]. FTD was secondarily diagnosed in 15% of these ALS patients using previously established criteria [76]. FALS and SALS patients were negative for mutations in *C9orf72*, *SOD1*, *TARDBP* and *FUS*, which are the main ALS genes [58,77]. FALS and autopsied patients had also been systematically screened for rare ALS genes, including *ANG*, *DAO*, *MATR3*, *OPTN*, *SQSTM1*, *SS18L1*, *VAPB* and *VCP* [78,79,80,81,82]; however, patients carrying variants in these rare ALS genes were included in the present study. All participants signed a consent form for the research and protocols were approved by the Medical Research Ethics Committee of “Assistance Publique-Hôpitaux de Paris”. The 3 PFN1 coding exons were analysed by Sanger sequencing (Table S1).

### 4.2. Patient Lymphoblasts

Lymphoblastoid cell lines were generated by Epstein Barr virus transformation of peripheral blood mononuclear cells from the FALS patient carrying the M114T mutation, the SALS patient carrying the E117G variant and two healthy controls (at the Genethon cell bank, Evry, France). Lymphoblasts were grown in RPMI 1640 supplemented with 10% foetal bovine serum, 50 U/mL penicillin and 50 mg/mL streptomycin (Thermo Fisher Scientific, Courtaboeuf, France) renewed twice a week. For protein degradation assay, 1.5 × 10^6^ cells were treated with 200 µg/mL cycloheximide for 2, 4, 6, 8, 10 or 12 h. Controls cells (time point 0) received equivalent volume of dimethylsulfoxide (DMSO). For lysosomal degradation inhibition, 1.5 × 10^6^ cells were treated with 10 mM of NH_4_Cl for 24 h at 37 °C. For activation of conventional and/or alternative autophagy pathways, 1.5 × 10^6^ cells were incubated with 50 mM trehalose for 24 h or 20 µM etoposide for 18 h, respectively. Non-treated cells received an equivalent volume of water or DMSO, respectively. Although etoposide can induce cell toxicity, we did not observe cell death and the total content of protein between untreated and etoposide treated cells was comparable. All treatment reagents were from Sigma-Aldrich. For pellets, lymphoblasts were centrifuged 5 min at 900 g and rinsed in phosphate-buffered saline (PBS). Dry pellets were frozen at −80 °C.

### 4.3. Semi Quantitative RT-PCR

Total RNA was extracted from lymphoblasts and mouse spinal cord using the Trizol reagent (Thermo Fisher Scientific), subjected to RNase-free DNase treatment (Qiagen) and purified using RNeasy columns (Qiagen). First-strand cDNA synthesis was performed using the ThermoScript RT-PCR system (Thermo Fisher Scientific) according to the manufacturer’s instructions. Primer sequences and PCR conditions for amplification of these cDNAs are described in Table S1. PCR products were loaded on 1.5% agarose gels containing 10 mg/mL ethidium bromide that were run for 30 min at 100 V and exposed to UV. The signal intensity of the captured images was analysed with MultiGauge 3.0 software (Fuji Film).

### 4.4. Antibodies

Primary antibodies against GAPDH (D16H11, Cell Signaling Technology, Leiden, The Netherlands), HA (ab9110, Abcam, Amsterdam, The Netherlands), LC3B (NB100-2220, Novus Biologicals, Noyal Chatillon sur Seiche, France), MFN2 (M6319, Sigma-Aldrich, Saint Quentin Fallavier, France), PFN1 (ab124904, Abcam), RAB9 (11420-1-AP, ProteinTech, Manchester, UK) were made in rabbit. Antibody against HA (ab9134, Abcam) was made in goats, whereas those against Cytochrome C (556432 and 556433, BD Pharmingen, Le Pont de Claix, France) and RAB9 (ab2810, Abcam) were made in the mouse. Antibody against GFP was made in chicken (A10262, Thermo Fisher Scientific). Peroxidase-conjugated secondary antibodies were goat anti-mouse or anti-rabbit with minimal cross-reaction to human serum proteins (Jackson ImmunoResearch Laboratories, Cambridgeshire, UK). Fluorochrome-conjugated secondary antibodies (Alexa FluorTM 488, 555 or 641, Thermo Fisher Scientific) were goat anti-mouse IgG1 (for anti-CYCS), anti-mouse, -rabbit or -chicken (made in goat) or donkey anti-goat antibodies.

### 4.5. Neuropathology

Immunostaining for PFN1 was performed on three levels of the patient spinal cord (cervical thoracic and lumbar) and on the medulla oblongata, frontal isocortex, hippocampus and cerebellum (embedded in paraffin). After deparaffinization, 5 µm thick sections were immunolabeled with PFN1 antibodies in an automatic slide stainer (Benchmark^®^ XT Ventana^®^ staining system), the slides being pre-treated at 95 °C in CC1 (pH = 8) proprietary retrieval buffers (Ventana Medical Systems, Illkirch-Graffenstaden, France). The biotinylated secondary antibody was included in the detection kit (Ventana Medical Systems Basic DAB Detection Kit 250-001). The streptavidin-biotin-peroxidase complex was revealed by diamino-benzidine.

### 4.6. Immunoblotting

For immunoblots, samples were homogenized in 50 mM Tris-HCl pH 8, 150 mM NaCl, 1 mM MgCl_2_, cOmplete™ Mini EDTA-free protease inhibitor cocktail and PhosStop phosphatase inhibitors and incubated at 37 °C for 30 min with 0.5 U/µL Benzonase™ nuclease (all from Sigma-Aldrich). Sodium Dodecyl Sulfate (SDS) was added at a final concentration of 2% and cells were homogenized again. Protein extracts were centrifuged at 16,000 *g* for 10 min. Protein concentration of supernatants was estimated by the bicinchoninic acid assay (Sigma-Aldrich). Proteins (15 µg) were denatured 10 min at 80 °C, separated on NuPAGE^TM^ 4–12% Bis-Tris Gel (Thermo Fisher Scientific) and electrophoretically transferred to nitrocellulose membranes (PROTAN^TM^, Whatman Gmbh, Dassel, Germany). Membranes were saturated for 10 min and incubated for 3 h with primary antibodies in PBS, 5% fat free milk, 0.1% Tween 20, followed by 1 hour of incubation with appropriate secondary antibodies. Signals were detected using ECL^TM^ Prime Western Blotting Detection Reagent (GE Healthcare SAS, Velizy-Villacoublay, France) and their intensity was analysed with MultiGauge 3.0 software (Fuji Film, Asnières-sur-Seine, France).

### 4.7. Plasmid and Lentiviral Constructs

All procedures using recombinant plasmids, lentiviruses and transgenic animals received approval from the French authorities for the use of genetically modified organisms. Three cDNAs of PFN1 (PFN1^WT^, PFN1^M114T^ and PFN1^E117G^) fused with a hemagglutinin tag (HA) at the N-terminus were ordered from ATUM (USA). They were subcloned under the control of the 378 bp ubiquitously expressed human Ubiquitin C (UBC) promoter [83] in lentiviral plasmids by the Gateway LRTM (Thermo Fisher Scientific) cloning method. The three corresponding lentiviruses (PFN1^WT^, PFN1^M114T^, PFN1^E117G^) were produced by the iVector core facility (ICM, Paris) using calcium chloride cotransfection of 3 plasmids (encoding the transgene, the encapsidation plasmid p8.7 or the vesicular stomatitis virus envelope) in HEK293T [84]. The vector titration was determined using the quantification of the p24 protein by ELISA (HIV-p24 antigen assay kit, Beckman Coulter). Five other PFN1 plasmid constructs expressing human PFN1 cDNA (ePFN1^WT^, ePFN1^G71V^, ePFN1^M114T^, ePFN1^E117G^, ePFN1^G118V^) fused with enhanced Green Fluorescent Protein (eGFP) tag at the N-terminus under the control of the human cytomegalovirus (CMV) immediate early enhancer and promoter were synthetized by ATUM (Newark, CA, USA) and used to transfect NSC-34 motor neuron cell line.

### 4.8. Cell Transfection

HEK293T human embryonic kidney cell line was obtained from ATCC and the Mouse Motor Neuron-Like Hybrid Cell Line NSC-34 was from Tebu-bio [85]. They were grown in Dulbecco’s modified Eagle’s medium, DMEM, containing 10% foetal calf serum and 0.1 mM nonessential amino acids (all from Life Technologies, Thermo Fisher Scientific). HEK293T cells were transfected using the calcium chloride method. NSC-34 cells were transfected using the nucleofection/electroporation AMAXA^TM^ technology (Lonza, Verviers, Belgium) according to manufacturer’s recommendations (Kit V, T-027 program) on a Nucleofector II^TM^ apparatus. Cells were harvested or fixed (4% paraformaldehyde prepared in PBS for 10 min) 24 to 48 h post-transfection. Cell culture experiments were performed at CELIS (ICM, Paris, France).

### 4.9. Mitochondrial Activity Assays

To measure ATP production, we used the Seahorse Bioanalyser XFe (Agilent Technologies, Les Ulis, France) as recommended by the manufacturer [86]. To assess mitochondrial activity, we also used the modified MTT (3-(4,5-dimethylthiazol-2-yl)-2,5-diphenyltetrazolium bromide) assay. This test measures the reduction of the yellow soluble tetrazolium salts into purple insoluble formazan blue crystals by succinate dehydrogenase mitochondrial enzyme activity [87]. Tetrazolium salt was added to each well at a final concentration of 0.5 mg/mL for 4 h. A solution of propanolol-2 containing 8% 1M HCl was added to the cultures, and cell dishes were agitated for 10 min until complete dissolution of the formazan blue crystals. Absorption values were immediately determined at 540 nm.

### 4.10. Transgenic Mice Establishment and Housing

The transgenic mice were established by lentiviral transgenesis performed by the UMS 28 small animal phenotype core facilities (Sorbonne Universities, Paris, France) according to a procedure previously detailed [51]. This strategy is based on lentiviral vectors injection into the perivitellin space of oocytes allowing sufficient numbers of transgenic founder animals to be obtained and directly analysed without the need of generating strains. Mice were genotyped from tail DNA at two time points (just after birth and before euthanasia). To avoid the amplification of the endogenous mouse Pfn1, the forward primer was designed on the HA tag of the transgene and the reverse primer was designed on the lentiviral sequence. To verify the quality of the DNA an amplification of the mouse Sod1 was performed. Primer sequences and PCR conditions are described in Table S1. Mice were housed in an animal facility whose temperature and humidity was controlled and were handled by trained personnel. Two (minimum) to 5 (maximum) mice were placed in each cage with cotton nests to enrich the environment of the animals.

### 4.11. Behavioural Tests

Several behavioural tests were performed weekly from the age of 3 months for the four groups of mice: PFN1^WT^, PFN1^M114T^, PFN1^E117G^ and non-transgenic mice (C57BL/6). The grip strength was assessed on forelimbs and hindlimbs using a grip strength meter (Bioseb, Vitrolles, France). This test determines the maximal force developed by a mouse, that has grasped the specially designed grid (for front paws) or bar (for back paws) when the operator tries to pull it out. The force achieved when the animal releases the grid (or the bar) is recorded. Three measures were recorded at each time point and the mean of the score was calculated. The Rotarod test measured the time (and maximum speed) during which the animal keeps on walking on an accelerating (from 4 to 40 rpm in 5 min) rotating wheel (Bioseb) and tests motor coordination and fatigability. For traction, each mouse was suspended by its front legs on a tight rope (of 5 mm diameter) and the time (arbitrarily limited to 30 s) used to put one hind leg onto the rope was measured [88]. The maximum score (30 s) was attributed in case the mouse fell from the rope.

### 4.12. Recordings of ElectroMyoGraphy and Compound Muscle Action Potential (CMAP)

All recordings were realized by the PHENO-ICMice core facilities (ICM, Paris) using a standard EMG apparatus (Neuro-Mep-Micro, Neurosoft, Russia). Following deep analgesic administration (0.1 mg/kg buprenorphine), animals were anesthetized by intraperitoneal injection of 0.5 µg/g Xylazine and 1 µg/g Ketamine and placed on a warming pad. To prevent the risk of eye damage: the application of an ophthalmic gel (Ocrygel) was applied at the time of recordings and a vitamin A ointment when waking up. The bilateral hind limbs of all animals were measured and averaged. Spontaneous activities of gastrocnemius were measured with a concentric electrode (Technomed, TE/B50600-001, Maastricht, The Netherlands). The signal was band pass 20 Hz to 10 KHz. The semi-quantitative assessment of spontaneous activity severity was performed using a 5-point ordinal scale (0: no spontaneous activities, 1: very rare, 2: rare, 3: common and 4: numerous) as previously described [89]. CMAP was recorded by an active recording needle electrode inserted distal to the knee joint over the proximal portion of the gastrocnemius and a reference recording needle electrode inserted over the Achilles tendon (Spesmedica, MN3512P150, Genova, Italy). The sciatic nerve was stimulated proximally at the sciatic notch and distally at the ankle with supramaximal stimuli of 0.2 ms through a needle electrode. Distal Motor Latency, expressed in ms, was determined by the time between the stimulus and the time to onset of a negative peak in the CMAP. For each measurement, a monopolar needle was inserted subcutaneously at the base of the tail to ground system. After these recordings, analgesic (Buprenorphine) and non-steroidal anti-inflammatory (Ketoprofen) were administered upon awaking (and repeated twice a day for 2 days).

### 4.13. Mouse Tissue Collection and Preparation

Mice were euthanized at 17 months of age by intraperitoneal administration of 0.5 µg/g Xylazine/ 1 µg/g Ketamine, followed by transcardial perfusion of PBS. Spinal cord, brain, gastrocnemius muscles, nerves (sciatic and hypoglossal nerves), kidney, lung, liver, and spleen were collected. For molecular analysis, half of the samples were rapidly frozen in liquid nitrogen and kept frozen at −80 °C until further use. For immunohistological analysis, the other half of the tissues was fixed for 24 h in PBS containing 4% paraformaldehyde, placed in PBS containing 30% sucrose for 48 h, frozen and cut on a cryostat (20 µm sections, on slides) at the Histology core facility (ICM, Paris, France).

### 4.14. Immunohistological Analyses

Fixed cells (HEK293T and NSC-34 on glass coverslips) and mouse tissue sections were incubated with a permeabilisation solution containing PBS, gelatin (0.1%), goat/donkey serum (10%) and Triton (0.3%), for 30 min. The primary antibody was diluted at appropriate concentrations in the permeabilisation solution and incubated overnight. After 3 washes in PBS, the slides were incubated for 1 h with corresponding secondary fluorochrome conjugated antibody (Alexa Fluor, Invitrogen, Thermo Fisher Scientific) diluted at 1:250. After 3 washes in PBS, the slides were incubated 10 min with DAPI and mounted with moviol solution.

### 4.15. Cell Counting and Signal Quantification

After double immunofluorescence, the number of HA expressing motor neurons (detected by anti-HA antibody) which do or do not present mitochondrial sparse distribution (detected using anti-cytochrome C antibody) were counted for each mouse in a blind manner on every 8th section of the lumbar spinal cord (70 to 130 HA-positive motor neurons were recorded for each mouse). For quantification of HA signal intensity in motor neurons, sections were stained for the HA epitope tag. After staining, images of the spinal cord were acquired using Axio Scan Z1 scanner (Zeiss, Oberkochen, Germany). The motor neurons in the ventral horn of the spinal cord were measured for their fluorescence intensity using Fuji software. Measures were realized from every 8th 20 μm sciatic nerve section, corresponding to a total of 5–7 sections per animal. The ventral horn was circled, a common detection threshold was set up for every section and the average fluorescence intensity was calculated. For quantification of RAB9 and CytC immunofluorescence signal intensity, triple immunofluorescence stainings were performed using anti-RAB9A (detected in far red), anti-CYCS (detected in red) and anti-GFP (detected in green). Each Cell expressing GFP was circled, intensity of red and far red was recorded. A total number of 20 to 70 transfected cells were recorded for each plasmid construct.

### 4.16. Electronic Microscopy Analysis

Samples were washed 3 times in 0.1M sodium cacodylate buffer (pH 7.4) and post-fixed with 1% osmium tetroxide solution diluted in cacodylate buffer for 1 h at 4 °C. After 3 washes with water, they were incubated for 2 h in 2% uranyl acetate solution and dehydrated in a graded series of ethanol solutions (50%, 70%, 80%, 90% and 100%). Final dehydration was performed twice in acetone for 20 min. Samples were then progressively infiltrated with Epon 812 epoxy resin (EMS, Souffelweyersheim, France) in 2 steps: overnight in a 1:1 mixture of Epon and acetone in an airtight container at 4 °C and 2 h at room temperature in pure Epon. They were placed in molds with fresh resin in a dry oven at 56 °C for 48 h. Blocs were cut in 0.5 µm semi-thin sections with an ultramicrotome EM UC7 (Leica, Leica Microsystemes SAS, Nanterre, France). Sections were stained with 1% toluidine blue solution in borax buffer 0.1 M. Ultra-thin sections (70 nm thick) were collected on nickel grids (immunoelectron microscopy) and contrasted with Reynold’s lead citrate for 7 min [90]. Observations were made with a Hitachi HT7700 electron microscope (Elexience, Verrière-le-Buisson, France) operating at 70 kV. Electron micrographs were captured with an AMT XR41B camera (2048 × 2048 pixels). Normal (examples showed in Figure S7A,B) and abnormal (examples presented in Figure S8C–G) mitochondria were recorded (70 to 230 mitochondria per mice were observed in a blinded manner for 2 mice of each genotype) to calculate the percentage of abnormal mitochondria.

### 4.17. Statistical Analyses

Normality of the sample distribution was assessed using the Shapiro–Wilk test before using parametric analyses. Analysis of variance (ANOVA) or a Kruskal–Wallis test (for non-parametric multigroup comparison) were used to compare protein densitometry analyses, mRNA levels, spontaneous activity scores, HA positive mouse motor neurons with mitochondrial abnormalities or immunofluorescence intensities. Then, if this analysis showed a statistical difference, the post hoc (multiple comparisons) Tukey’s test (or Dunn’s test for non-parametric analyses) was used to compare groups 2 by 2. For kinetic and behavioural curves, repeated-measures ANOVA was used with post-hoc Bonferroni tests. Statistical analyses were performed using GraphPad Prism software (v.6).

## Data Availability

All datasets generated or analysed during the study are available upon request.

## Supplementary Materials

The following supporting information can be downloaded at: www.mdpi.com/article/10.3390/ijms23105694/s1.
