# Supplementary material for: The Amyotrophic Lateral Sclerosis M114T PFN1 Mutation Deregulates Alternative Autophagy Pathways and Mitochondrial Homeostasis
Source: Int J Mol Sci. 2022 May 19;23(10):5694. doi: 10.3390/ijms23105694 (PMC9143529; doi:10.3390/ijms23105694)
Supplement: Supplementary file 1 [file ijms-23-05694-s001.zip › ijms-1727092-supplementary.pdf]

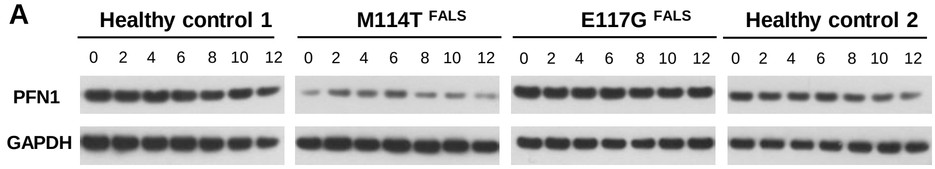

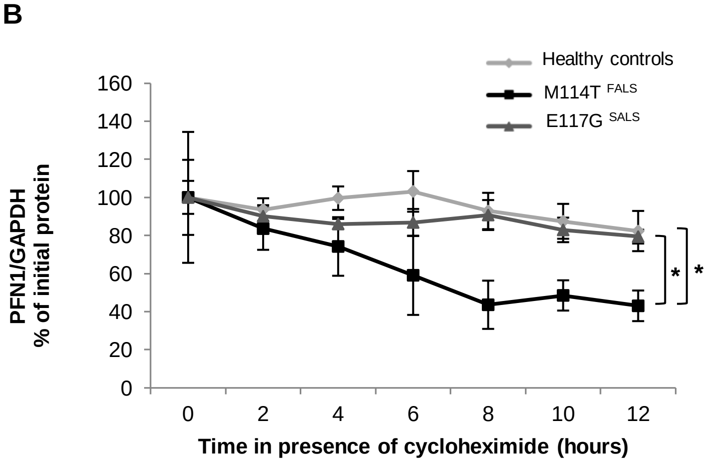

Figure S1. Protein instability of the M114T mutant form of PFN1. (A) Patient lymphoblasts were treated with cycloheximide (an inhibitor of translation) and harvested at different time points (0, 2, 4, 6, 8, 10 and 12 hours). Immunoblot analysis of protein extracts from lymphoblasts of healthy controls and ALS patients carrying the M114T mutation or the E117G variant using anti-PFN1 and GAPDH antibodies. (B) Kinetic curves showing the rate of degradation of PFN1 in lymphoblasts of healthy controls (light grey), M114T patient (black) and E117G patient (dark grey) in the presence of cycloheximide. Results are means +/- SEM of 4 experiments and are presented as percentage of initial protein. *p<0.05.

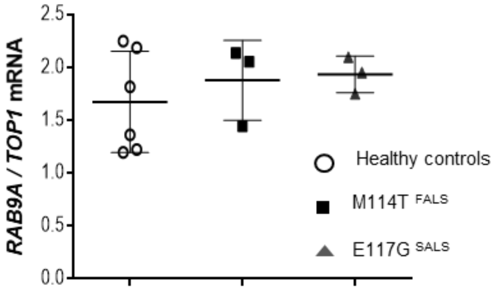

Figure S2. Analysis of RAB9 mRNA levels in patient lymphoblasts. Semiquantitative RT-PCR for *RAB9A* mRNA extracted from patients' lymphoblasts: two healthy controls in white circles, M114T patient in black squares and E117G patient in grey triangles. The levels of *RAB9A* mRNA were normalized to that of DNA topoisomerase 1 (*TOP1*). Results are means +/- SEM of 3 independent experiments.

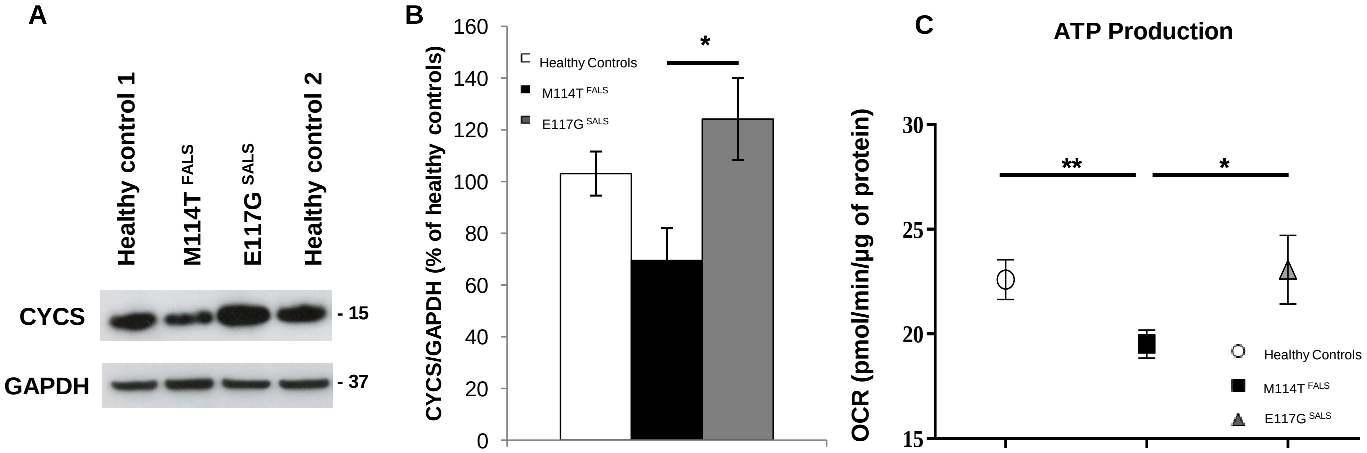

Figure S3. Deregulation of additional mitochondrial markers in M114T patient lymphoblasts. (A) Anti-CYCS antibody was used to quantify mitochondria levels in lymphoblast protein extracts. (B) Densitometry analyses of CYCS protein levels were recorded for healthy controls (white), M114T patient (black) and E117G patient (grey), normalized to GAPDH levels and presented as percentage of healthy controls. (C) For mitochondrial ATP production, OCR (oxygen consumption rate) for controls (white circles), M114T patient (black squares) and E117G patient (grey triangles) was measured in lymphoblasts by Seahorse Bioanalyser. Results are means +/- SEM of 4 independent experiments. *p<0.05; **p<0.01.

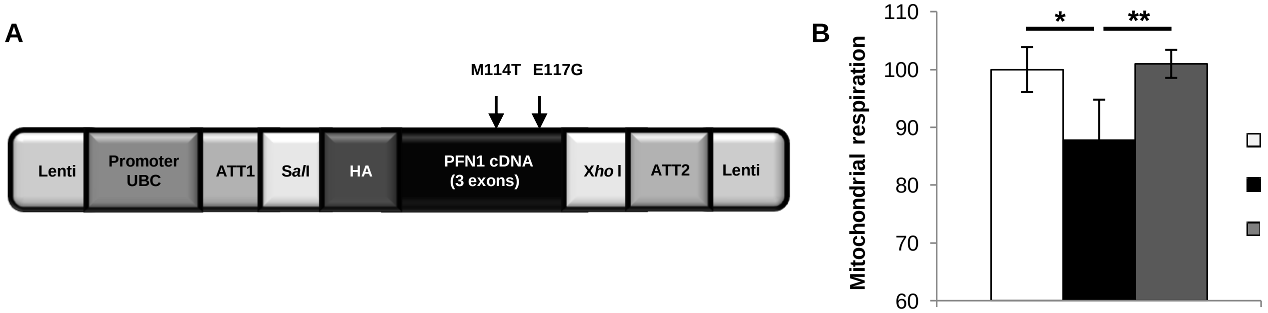

Figure S4. Analysis of mitochondrial respiration in cells transfected with HA-tagged PFN1 lentiviral plasmid constructs. (A) Schematic representation of lentiviral plasmid constructs designed for lentiviral transgenesis to overexpress PFN1 fused to a hemagglutinin (HA) tag under the control of the ubiquitously expressed human Ubiquitin C (UBC) promoter. (B) Mitochondrial respiration was measured in HEK293T expressing PFN1^{WT} (white), PFN1^{M114T} (black) and PFN1^{E117G} (grey) plasmid constructs using the MTT assay. Results are means +/- SEM for 4 independent experiments. *p<0.05; **p<0.01.

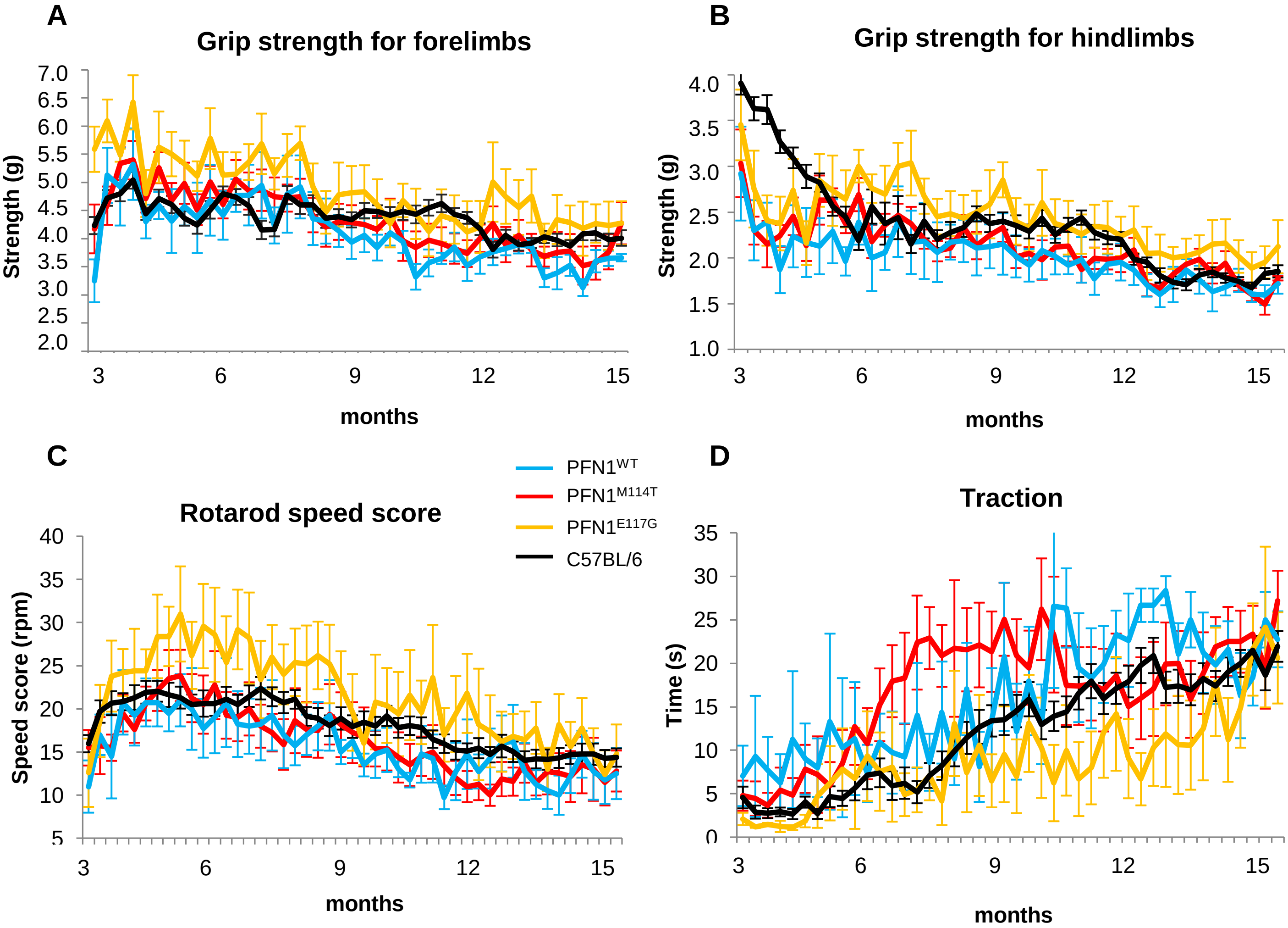

Figure S5. Behavioral analysis of PFN1 mice.

Behavioral tests were performed in mice once a week from the age of 3 months to evaluate muscle strength and observe the possible onset of paralysis. Curves represent the performances of the forelimb (A) or hindlimb (B) grip strength, the rotarod (C) and the traction test (D) for the PFN1WT (blue, n=4), PFN1M114T (red, n=8), PFN1E117G (orange, n=5) and C57BL/6 (black, n=38) mice.

A

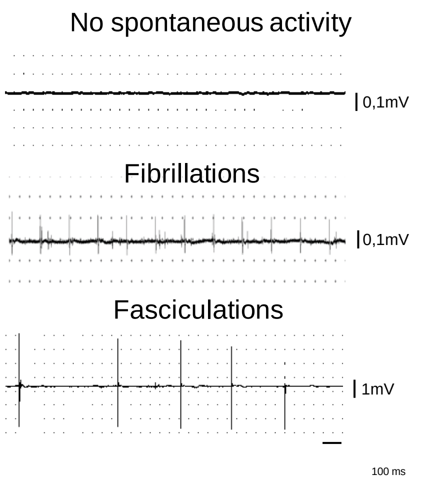

B

	11 months	17 months
**PFN1^{WT}**	0/4	2/4 (50%)
**PFN1^{M114T}**	0/8	6/6 (100%)
**PFN1^{E117G}**	0/5	2/5 (40%)
**C57BL/6**	0/4	7/25 (28/%)

C

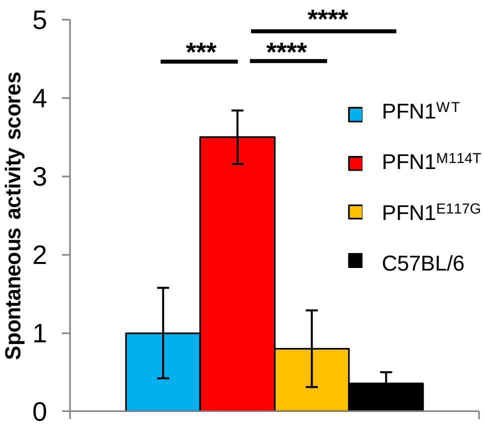

D

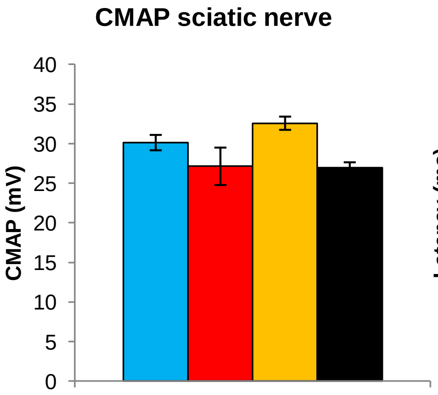

E

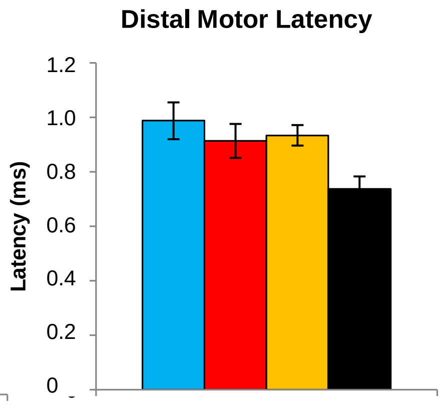

Figure S6. EMG recordings in the sciatic nerve of PFN1 mice.
(A) Examples of EMG profiles recorded in a mouse gastrocnemius muscle at the age of 17 months with no spontaneous activity (upper panel), fibrillations (middle panel) or fasciculations with a larger amplitude (lower panel). (B) Proportion of mice with spontaneous activities (fibrillations and/or fasciculations) at 11 and 17 months. (C) Comparison of the severity score for the spontaneous activities detected at 17 months for PFN1^{WT} (blue, n=4), PFN1^{M114T} (red, n=6), PFN1^{E117G} (orange, n=5) and C57BL/6 (black, n=25) mice. Evoked potentials were recorded at 17 months in the sciatic nerve. (D) Means +/- SEM of the Compound Muscle Action Potentialss (CMAP) and (E) the distal motor latency. ***$p<0.005$; ****$p<0.001$.

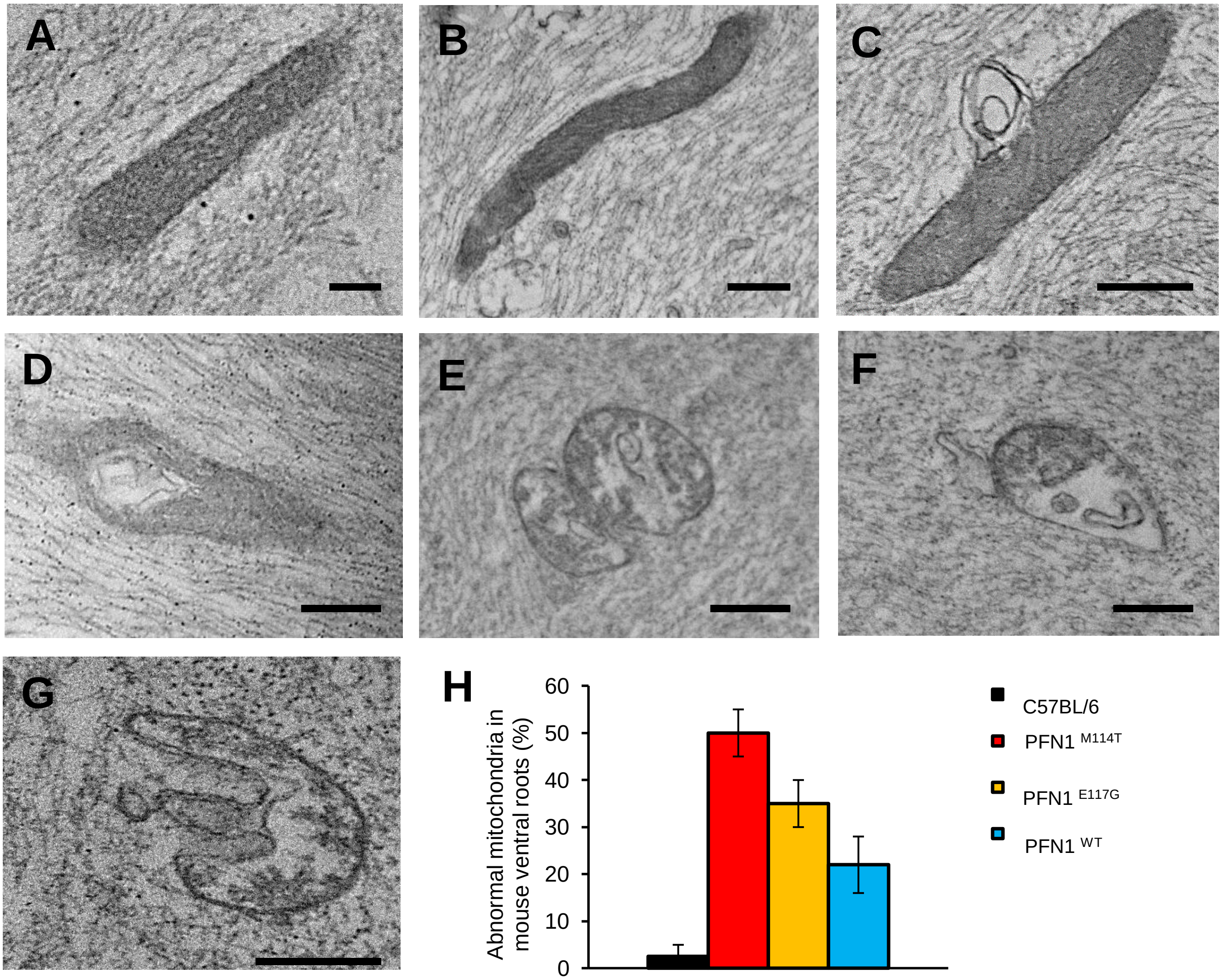

Figure S7. Mitochondrial morphology observed by electron microscopy in mouse ventral root motor axons.
(A) Electron micrographs of mitochondria observed in ventral root motor axons of C57BL/6, (B-C) $PFN1^{WT}$, (D) $PFN1^{E117G}$ and (E-G) $PFN1^{M114T}$ mice. (A) Examples of healthy mitochondria with dark matrix and cristae membranes in C57BL/6 and (B) in $PFN1^{WT}$. (C) Representative images showing examples of mitochondria remodeling with membrane vesicles in close apposition to the outer mitochondrial membrane in $PFN1^{WT}$ or (D) engulfing cytosolic components in $PFN1^{E117G}$ mice. (E-G) Examples of degenerative mitochondria in $PFN1^{M114T}$ mice with loss of cristae structure and clear matrix, (E-F) engulfing vesicles or (G) with aberrant shape. Bar: 1 µm. (H) Percentage of abnormal mitochondria were recorded from 70 to 230 mitochondria observed in 2 mice of each genotype.

Table S1. Clinical data of PFN1 patients.

Variant	Patient	Sexe	Age of onset (y)	Age of death (y)	Onset	Evolution (m)	Observation
**M114T**	FALS 1	M	50	53	distal lower limb (left)	28	
	FALS 2	F	49	50	distal lower limb (left)	12	
**E117G**	SALS 1	M	54	NA	cervical	NA	
	SALS 2	F	46	50	distal upper limb (right)	52	*ANG* (K17I) variant

NA: not available

Table S2. Primers and PCR conditions used for human sequencing, cDNA amplification and mouse genotyping.

	Target	Forward primer	Reverse primer	Annealing temperature	Number of cycles	fragment size (pb)
**Human sequencing**	*PFN1* exon1	GCGAGGGCTGCTGCACAGCGA	CGCTTCCAGGGCAAGCACCCAGT	60	35	410
	PFN1 exon2	GGAATCTTGGTGCACTGACTAACT	AAGCACCCTCAAGATTACCAGAA	60	35	320
	PFN1 exon3	GGGAGAGATGAGGTTGGGTTACA	GGTTTGTGTGTGTATGGGGAGGA	TD(70-60)	35	285
**cDNA amplification in lymphoblasts**	*PFN1*	CAACCTCATGGCGGACGGGAC	GCCAACCAGGACACCCACCTCA	60	21	133
	RAB9A	ATAAAGATTTGGAAGTGGATGGA	AAGTAAGCAGGCAGCAGTCAG	60	22	123
	TOP1	AATGCCTCCATCACGCTACAG	GGCACGGTTATAAGAAAGGATCTT	62	25	90
**Mouse genotyping**	*PFN1* transgenes	TACCCATACGATGTTCCAGAT	TAAAGGTACCGTCGAGACTAGTAGGTAT	56	35	714
	mSOD1	GTTACATATAGGGGTTTACTTCATAATCTG	CAGCAGTCACATTGCCCAAGGTCTCCAACAT	56	35	873
**cDNA amplification in mouse tissue**	*HA tagged-PFN1*	TACCCATACGATGTTCCAGAT	AGTCCTTGTAGCCCACGAT	56	25	106
	Gapdh	GAACATCATCCCTGCATCCA	CCAGTGAGCTTCCCGTTC	60	22	78

TD: touch-down.
